# Supplementary material for: A Likelihood Approach for Real-Time Calibration of Stochastic Compartmental Epidemic Models
Source: PLoS Comput Biol. 2017 Jan 17;13(1):e1005257. doi: 10.1371/journal.pcbi.1005257 (PMC5240920; doi:10.1371/journal.pcbi.1005257)
Supplement: S1 File — (TAR.GZ) [file pcbi.1005257.s014.tar.gz › HSPH_Online-SI-Revision/Additions/IPoi10/n100-extreme-SEITR/n100-extreme-SEITR_BENCH2_table4.pdf]

1	newly 3 weeks spec	1057.	2375.03	{1049., 4945.}	127.141	0.292769	newly 3 weeks cumul	5452.	9344.12	{4316., 18821.}	80.2258	0.216187	newly 5 weeks spec	382.	1381.09	{603., 2959.}	261.541	0.48022	newly 5 weeks cumul	6457.	12541.	{5717., 25518.}	99.1484	0.246871	newly 47 weeks cumul	7014.	16668.7	{7509., 34164.}	139.206	0.313742
2	newly 3 weeks spec	909.	2532.76	{1313., 4200.}	178.632	0.401269	newly 3 weeks cumul	5060.	12034.7	{6531., 19477.}	137.971	0.339413	newly 5 weeks spec	300.	1074.85	{573., 1778.}	258.284	0.50832	newly 5 weeks cumul	5879.	14756.9	{7980., 23987.}	151.01	0.360826	newly 54 weeks cumul	6291.	16678.6	{8924., 27103.}	165.118	0.383492
3	newly 3 weeks spec	1244.	947.775	{430., 1562.}	43.8024	0.243303	newly 3 weeks cumul	6664.	5363.06	{2523., 8597.}	41.8027	0.222907	newly 5 weeks spec	463.	315.366	{144., 495.}	45.5511	0.27275	newly 5 weeks cumul	7872.	6223.04	{2936., 9987.}	42.0907	0.227487	newly 56 weeks cumul	8546.	6648.39	{3117., 10652.}	42.2726	0.231379
4	newly 3 weeks spec	1162.	1667.66	{842., 2669.}	57.6478	0.174457	newly 3 weeks cumul	6474.	8015.75	{4115., 13087.}	44.2269	0.152066	newly 5 weeks spec	412.	697.926	{320., 1102.}	76.6598	0.206884	newly 5 weeks cumul	7613.	9783.79	{5036., 15956.}	47.0387	0.156043	newly 54 weeks cumul	8186.	11023.5	{5617., 18117.}	50.824	0.161936
5	newly 3 weeks spec	334.	873.958	{431., 1504.}	161.664	0.354798	newly 3 weeks cumul	2304.	5210.04	{2584., 9332.}	127.025	0.299217	newly 5 weeks spec	78.	266.153	{130., 479.}	241.222	0.469568	newly 5 weeks cumul	2553.	5962.15	{2952., 10725.}	134.229	0.311774	newly 56 weeks cumul	2676.	6284.17	{3128., 11315.}	135.53	0.31391
6	newly 3 weeks spec	210.	1528.82	{635., 3184.}	628.007	0.771619	newly 3 weeks cumul	1688.	8194.78	{3566., 16645.}	385.473	0.605751	newly 5 weeks spec	40.	538.985	{225., 1129.}	1247.46	1.0354	newly 5 weeks cumul	1825.	9648.54	{4145., 19653.}	428.687	0.641108	newly 55 weeks cumul	1849.	10412.9	{4457., 21228.}	463.164	0.667564
7	newly 3 weeks spec	1990.	2140.2	{1165., 3411.}	35.0055	0.139686	newly 3 weeks cumul	8888.	8575.81	{4751., 13616.}	32.3695	0.144861	newly 5 weeks spec	982.	1233.97	{649., 1927.}	43.1231	0.14632	newly 5 weeks cumul	11272.	11436.2	{6274., 18186.}	33.2758	0.141341	newly 47 weeks cumul	13516.	15078.4	{8149., 24196.}	35.8753	0.137874
8	newly 3 weeks spec	1262.	2592.45	{1449., 4069.}	106.779	0.262484	newly 3 weeks cumul	6258.	11815.6	{6972., 18914.}	90.1046	0.244459	newly 5 weeks spec	510.	1186.35	{584., 1716.}	133.688	0.278658	newly 5 weeks cumul	7632.	14775.6	{8477., 23246.}	94.9588	0.248224	newly 54 weeks cumul	8594.	16991.4	{9535., 26281.}	99.0944	0.250538
9	newly 3 weeks spec	351.	896.	{405., 1697.}	155.923	0.337887	newly 3 weeks cumul	2373.	5373.3	{2542., 10282.}	127.588	0.293478	newly 5 weeks spec	96.	277.478	{125., 555.}	189.04	0.387662	newly 5 weeks cumul	2626.	6144.59	{2850., 11801.}	134.903	0.306035	newly 56 weeks cumul	2718.	6482.65	{2978., 12432.}	139.301	0.31347
10	newly 3 weeks spec	2417.	1117.44	{575., 1885.}	55.7876	0.39129	newly 3 weeks cumul	10500.	5216.45	{2699., 8887.}	52.9092	0.359563	newly 5 weeks spec	1167.	508.56	{255., 844.}	58.2565	0.41898	newly 5 weeks cumul	13414.	6483.07	{3350., 11053.}	54.02	0.371355	newly 52 weeks cumul	16196.	7501.29	{3898., 12768.}	55.6351	0.389012
11	newly 3 weeks spec	2013.	2596.09	{1334., 4651.}	47.8913	0.161763	newly 3 weeks cumul	9231.	11037.4	{6031., 19670.}	41.5297	0.151606	newly 5 weeks spec	1003.	1324.64	{659., 2421.}	51.1827	0.168462	newly 5 weeks cumul	11702.	14224.1	{7660., 25521.}	43.1073	0.154612	newly 51 weeks cumul	13920.	17320.5	{9146., 31284.}	45.1458	0.158266
12	newly 3 weeks spec	1233.	2638.83	{1395., 4335.}	115.869	0.285775	newly 3 weeks cumul	6317.	11804.5	{6390., 18991.}	89.8016	0.240846	newly 5 weeks spec	542.	1234.08	{589., 1994.}	128.853	0.303255	newly 5 weeks cumul	7681.	14849.6	{7925., 23923.}	95.8749	0.251135	newly 52 weeks cumul	8581.	17384.2	{9153., 28092.}	104.781	0.267311
13	newly 3 weeks spec	1573.	1577.07	{808., 2660.}	40.8236	0.176294	newly 3 weeks cumul	7548.	7433.1	{3948., 12365.}	38.5529	0.169691	newly 5 weeks spec	706.	696.577	{348., 1203.}	40.4122	0.177723	newly 5 weeks cumul	9330.	9179.87	{4822., 15312.}	38.8661	0.171113	newly 53 weeks cumul	10733.	10535.1	{5546., 17554.}	39.1145	0.172508
14	newly 3 weeks spec	2628.	2180.58	{1115., 4043.}	40.1148	0.201414	newly 3 weeks cumul	11099.	8495.	{4499., 15482.}	39.9429	0.213898	newly 5 weeks spec	1313.	1306.67	{654., 2447.}	40.6194	0.171935	newly 5 weeks cumul	14282.	11494.4	{5987., 21070.}	39.8451	0.205329	newly 46 weeks cumul	17443.	15670.6	{7883., 29028.}	39.406	0.184948
15	newly 3 weeks spec	1421.	2705.44	{1450., 4439.}	92.5735	0.23961	newly 3 weeks cumul	6948.	10330.1	{5822., 16933.}	56.2826	0.170621	newly 5 weeks spec	621.	1653.09	{827., 2645.}	166.198	0.358722	newly 5 weeks cumul	8459.	14097.4	{7725., 23077.}	71.2359	0.19936	newly 47 weeks cumul	9505.	19412.8	{10252., 31542.}	105.456	0.26262
16	newly 3 weeks spec	1685.	2316.51	{1125., 4432.}	62.3384	0.186666	newly 3 weeks cumul	8356.	9500.67	{4841., 18333.}	51.1698	0.189319	newly 5 weeks spec	644.	1243.81	{562., 2459.}	99.4134	0.225824	newly 5 weeks cumul	10017.	12456.4	{6241., 24227.}	55.445	0.185655	newly 50 weeks cumul	11020.	15657.1	{7703., 30556.}	63.4316	0.183889
17	newly 3 weeks spec	244.	1688.46	{814., 3039.}	591.991	0.760584	newly 3 weeks cumul	2213.	9176.34	{4583., 16136.}	314.656	0.551461	newly 5 weeks spec	41.	572.246	{258., 970.}	1295.72	1.05957	newly 5 weeks cumul	2358.	10728.8	{5316., 18846.}	354.995	0.589328	newly 56 weeks cumul	2381.	11495.6	{5652., 20161.}	382.806	0.613853
18	newly 3 weeks spec	946.	2246.72	{922., 4553.}	139.608	0.26069	newly 3 weeks cumul	4981.	10428.8	{4909., 21703.}	110.853	0.25163	newly 5 weeks spec	314.	1038.14	{324., 1714.}	231.695	0.297586	newly 5 weeks cumul	5835.	13023.9	{5794., 27570.}	124.557	0.260786	newly 55 weeks cumul	6316.	14725.	{6303., 30018.}	134.503	0.265005
19	newly 3 weeks spec	2246.	3733.93	{1903., 5728.}	72.2764	0.203782	newly 3 weeks cumul	9933.	14813.6	{8033., 22180.}	56.6767	0.1763	newly 5 weeks spec	1136.	2064.21	{993., 3099.}	87.3478	0.228044	newly 5 weeks cumul	12584.	19668.2	{10419., 29529.}	63.1671	0.188491	newly 50 weeks cumul	14784.	25083.9	{12929., 37746.}	74.5596	0.20894
20	newly 3 weeks spec	688.	1553.7	{836., 2870.}	126.014	0.308935	newly 3 weeks cumul	3837.	8209.57	{4611., 14457.}	114.323	0.289673	newly 5 weeks spec	219.	564.73	{308., 1008.}	157.867	0.363794	newly 5 weeks cumul	4426.	9709.51	{5420., 17108.}	119.663	0.299471	newly 55 weeks cumul	4693.	10539.1	{5814., 18567.}	124.75	0.308678
21	newly 3 weeks spec	566.	1593.98	{859., 2878.}	181.622	0.394731	newly 3 weeks cumul	3401.	8845.71	{4855., 15839.}	160.091	0.365483	newly 5 weeks spec	156.	523.586	{277., 925.}	235.632	0.468737	newly 5 weeks cumul	3828.	10295.8	{5617., 18473.}	168.959	0.379148	newly 56 weeks cumul	3988.	11022.6	{6013., 19825.}	176.394	0.390553
22	newly 3 weeks spec	1150.	1223.42	{606., 2058.}	47.1173	0.180988	newly 3 weeks cumul	5834.	5621.32	{2782., 9705.}	44.8112	0.192851	newly 5 weeks spec	400.	553.076	{270., 907.}	59.0038	0.171	newly 5 weeks cumul	6931.	6998.36	{3443., 12028.}	45.6478	0.186763	newly 52 weeks cumul	7573.	8120.29	{3998., 13936.}	47.1438	0.180772
23	newly 3 weeks spec	361.	1680.61	{865., 2602.}	365.542	0.615813	newly 3 weeks cumul	2348.	8127.74	{4320., 12780.}	246.156	0.491512	newly 5 weeks spec	79.	698.246	{345., 1120.}	783.856	0.892173	newly 5 weeks cumul	2588.	9912.38	{5234., 15603.}	283.013	0.534536	newly 53 weeks cumul	2670.	11169.3	{5835., 17579.}	318.328	0.572065
24	newly 3 weeks spec	617.	1917.91	{843., 3300.}	211.467	0.416653	newly 3 weeks cumul	3939.	9979.33	{4527., 16977.}	154.479	0.340536	newly 5 weeks spec	171.	705.766	{307., 1231.}	312.728	0.535833	newly 5 weeks cumul	4404.	11854.	{5363., 20211.}	169.969	0.363527	newly 55 weeks cumul	4602.	12886.9	{5821., 22083.}	180.736	0.37925
25	newly 3 weeks spec	1330.	3450.14	{1929., 4520.}	159.409	0.373052	newly 3 weeks cumul	7007.	14279.8	{8445., 18682.}	104.745	0.281125	newly 5 weeks spec	443.	1778.3	{981., 2288.}	301.421	0.554601	newly 5 weeks cumul	8227.	18565.1	{10844., 24151.}	125.891	0.318978	newly 51 weeks cumul	8870.	22827.3	{13175., 29600.}	157.354	0.372074
26	newly 3 weeks spec	920.	2960.63	{1534., 4535.}	221.808	0.454796	newly 3 weeks cumul	5136.	12025.8	{6513., 18195.}	134.148	0.325621	newly 5 weeks spec	345.	1602.	{800., 2486.}	364.348	0.6037	newly 5 weeks cumul	6057.	15810.3	{8348., 24015.}	161.025	0.369102	newly 49 weeks cumul	6590.	19970.7	{10458., 30622.}	203.046	0.430234
27	newly 3 weeks spec	2118.	1455.82	{687., 2183.}	51.7684	0.289445	newly 3 weeks cumul	9250.	6029.15	{2940., 9181.}	49.9253	0.291309	newly 5 weeks spec	1079.	788.712	{376., 1164.}	53.3027	0.281593	newly 5 weeks cumul	11803.	7898.09	{3795., 11951.}	50.4456	0.28823	newly 49 weeks cumul	14480.	9922.74	{4682., 14934.}	51.2769	0.287198
28	newly 3 weeks spec	196.	1110.97	{517., 1764.}	466.821	0.680042	newly 3 weeks cumul	1632.	6253.09	{3063., 9464.}	283.155	0.517243	newly 5 weeks spec	32.	370.703	{166., 569.}	1058.45	0.98644	newly 5 weeks cumul	1750.	7267.97	{3557., 10937.}	315.313	0.551237	newly 56 weeks cumul	1781.	7769.38	{3786., 11730.}	336.237	0.572038
29	newly 3 weeks spec	1912.	976.94	{523., 1613.}	50.7892	0.3406	newly 3 weeks cumul	9043.	4653.79	{2579., 7667.}	50.2661	0.335159	newly 5 weeks spec	849.	428.075	{234., 681.}	51.5233	0.350282	newly 5 weeks cumul	11162.	5728.52	{3149., 9474.}	50.4287	0.337098	newly 53 weeks cumul	12786.	6549.79	{3625., 10865.}	50.5401	0.338147
30	newly 3 weeks spec	1221.	1814.84	{825., 3004.}	68.2702	0.171219	newly 3 weeks cumul	6812.	9394.97	{4545., 16513.}	56.7266	0.165032	newly 5 weeks spec	428.	634.043	{250., 1027.}	72.6568	0.175511	newly 5 weeks cumul	7969.	11103.5	{5261., 19344.}	58.809	0.166394	newly 56 weeks cumul	8592.	11962.5	{5611., 20714.}	59.4097	0.16673
31	newly 3 weeks spec	1586.	2669.85	{1345., 4813.}	73.9386	0.205479	newly 3 weeks cumul	7252.	10319.4	{5330., 18333.}	53.8142	0.169384	newly 5 weeks spec	689.	1599.62	{776., 2998.}	132.205	0.311578	newly 5 weeks cumul	9024.	14000.1	{7081., 25248.}	63.4218	0.187073	newly 46 weeks cumul	10290.	19130.9	{9542., 34820.}	89.3246	0.236941
32	newly 3 weeks spec	2184.	2424.46	{1009., 4922.}	59.9868	0.224652	newly 3 weeks cumul	9583.	10524.8	{4873., 22014.}	53.9297	0.205568	newly 5 weeks spec	1094.	1150.68	{468., 2473.}	63.4993	0.250865	newly 5 weeks cumul	12124.	13353.3	{6087., 27982.}	55.8196	0.211419	newly 52 weeks cumul	14626.	15727.2	{7084., 33006.}	56.8414	0.220402
33	newly 3 weeks spec	643.	236.	{119., 424.}	63.7957	0.493394	newly 3 weeks cumul	3804.	1448.22	{718., 2583.}	62.5056	0.477075	newly 5 weeks spec	197.	72.254	{36., 123.}	63.8708	0.496197	newly 5 weeks cumul	4382.	1653.73	{820., 2885.}	62.8114	0.480836	newly 56 weeks cumul	4693.	1741.41	{865., 3019.}	63.3804	0.487922
34	newly 3 weeks spec	883.	1817.59	{890., 2864.}	109.06	0.256211	newly 3 weeks cumul	4731.	8058.25	{4074., 12831.}	77.2417	0.206287	newly 5 weeks spec	278.	855.695	{389., 1353.}	208.194	0.404818	newly 5 weeks cumul	5501.	10166.6	{5129., 16171.}	89.7291	0.227116	newly 52 weeks cumul	5915.	11948.9	{6000., 18956.}	105.138	0.25292
35	newly 3 weeks spec	239.	1514.72	{805., 2612.}	533.774	0.752803	newly 3 weeks cumul	1921.	8494.05	{4695., 14348.}	342.168	0.600569	newly 5 weeks spec	50.	505.175	{260., 926.}	910.351	0.952812	newly 5 weeks cumul	2064.	9875.25	{5416., 16686.}	378.452	0.634029	newly 56 weeks cumul	2096.	10552.8	{5731., 17872.}	403.475	0.655917
36	newly 3 weeks spec	2432.	4055.79	{1976., 6374.}	74.3844	0.205109	newly 3 weeks cumul	10891.	16624.6	{8575., 25917.}	60.5047	0.182859	newly 5 weeks spec	1211.	2097.32	{951., 3296.}	81.6285	0.210313	newly 5 weeks cumul	13782.	21668.4	{10912., 33923.}	65.0099	0.189953	newly 52 weeks cumul	16479.	26519.3	{13088., 41568.}	68.8714	0.195513
37	newly 3 weeks spec	1023.	2794.3	{1380., 4877.}	173.471	0.375798	newly 3 weeks cumul	5532.	12680.4	{6666., 22098.}	130.653	0.314225	newly 5 weeks spec	383.	1258.89	{614., 2294.}	228.692	0.44765	newly 5 weeks cumul	6497.	15816.5	{8214., 27686.}	144.379	0.335308	newly 53 weeks cumul	7036.	18285.3	{9347., 32167.}	160.363	0.359765
38	newly 3 weeks spec	915.	1977.13	{909., 3465.}	118.787	0.272484	newly 3 weeks cumul	5124.	8405.32	{4031., 14530.}	73.0547	0.196046	newly 5 weeks spec	323.	1001.89	{456., 1731.}	210.182	0.411332	newly 5 weeks cumul	5962.	10812.5	{5103., 18754.}	87.1833	0.219894	newly 50 weeks cumul	6391.	13193.5	{6141., 22889.}	109.875	0.260387
39	newly 3 weeks spec	1339.	2011.59	{964., 3487.}	64.1112	0.188569	newly 3 weeks cumul	6703.	9135.22	{4541., 15912.}	53.6755	0.172498	newly 5 weeks spec	464.	923.478	{406., 1636.}	103.542	0.256683	newly 5 weeks cumul	7906.	11423.3	{5568., 19869.}	58.9948	0.180192	newly 52 weeks cumul	8666.	13296.6	{6413., 23102.}	65.1721	0.189842
40	newly 3 weeks spec	1822.	669.072	{366., 999.}	64.1197	0.489054	newly 3 weeks cumul	8114.	3308.	{1787., 5106.}	60.9199	0.444393	newly 5 weeks spec	874.	279.377	{152., 433.}	68.5322	0.549549	newly 5 weeks cumul	10273.	4014.51	{2145., 6183.}	62.1455	0.46133	newly 53 weeks cumul	12291.	4508.14	{2391., 6900.}	64.1022	0.487527
41	newly 3 weeks spec	362.	1295.72	{778., 1783.}	257.934	0.519943	newly 3 weeks cumul	2774.	7293.93	{4438., 9886.}	162.939	0.387572	newly 5 weeks spec	74.	428.295	{246., 588.}	478.777	0.724976	newly 5 weeks cumul	2996.	8475.61	{5163., 11443.}	182.897	0.418876	newly 56 weeks cumul	3059.	9054.56	{5509., 12257.}	195.997	0.438433
42	newly 3 weeks spec	2612.	2302.92	{1306., 3735.}	34.805	0.166626	newly 3 weeks cumul	10679.	9209.18	{5388., 14951.}	33.4187	0.163736	newly 5 weeks spec	1374.	1321.	{732., 2208.}	35.1352	0.154423	newly 5 weeks cumul	13825.	12285.7	{7152., 20131.}	33.6017	0.160138	newly 47 weeks cumul	17141.	16229.1	{9280., 26774.}	33.842	0.151902
43	newly 3 weeks spec	2253.	1414.42	{637., 2515.}	50.4947	0.316593	newly 3 weeks cumul	10165.	6494.35	{2913., 11633.}	48.9342	0.30348	newly 5 weeks spec	1018.	643.79	{272., 1114.}	51.4117	0.323186	newly 5 weeks cumul	12779.	8095.56	{3577., 14458.}	49.4558	0.308366	newly 52 weeks cumul	15131.	9392.53	{4129., 16902.}	50.1471	0.316548
44	newly 3 weeks spec	1384.	2783.25	{1338., 5035.}	104.06	0.253304	newly 3 weeks cumul	6755.	12208.2	{6228., 21520.}	84.88	0.225235	newly 5 weeks spec	626.	1309.22	{606., 2390.}	112.444	0.261869	newly 5 weeks cumul	8295.	15437.6	{7733., 27349.}	89.9745	0.232695	newly 52 weeks cumul	9359.	18131.3	{8929., 32330.}	97.0007	0.244056
45	newly 3 weeks spec	1196.	2418.74	{1245., 4891.}	103.404	0.250725	newly 3 weeks cumul	6409.	11426.8	{6141., 22525.}	80.7017	0.211548	newly 5 weeks spec	536.	1032.01	{511., 2079.}	95.322	0.230741	newly 5 weeks cumul	7757.	14047.2	{7454., 27836.}	83.4708	0.215753	newly 54 weeks cumul	8640.	15902.2	{8421., 31562.}	86.2633	0.220509
46	newly 3 weeks spec	1149.	549.813	{247., 1009.}	59.6687	0.421339	newly 3 weeks cumul	6165.	3148.8	{1454., 5620.}	57.5854	0.393608	newly 5 weeks spec	377.	180.906	{86., 315.}	60.3946	0.422965	newly 5 weeks cumul	7203.	3646.95	{1666., 6472.}	57.8611	0.397074	newly 56 weeks cumul	7768.	3891.17	{1778., 6931.}	58.1824	0.401082
47	newly 3 weeks spec	1358.	1925.13	{898., 3175.}	55.5271	0.169224	newly 3 weeks cumul	7212.	8447.65	{4088., 14009.}	40.1718	0.144301	newly 5 weeks spec	550.	946.449	{431., 1633.}	79.1225	0.211529	newly 5 weeks cumul	8640.	10752.3	{5186., 17928.}	43.8844	0.148448	newly 51 weeks cumul	9536.	12876.	{6103., 21558.}	50.4163	0.159171
48	newly 3 weeks spec	874.	1885.	{838., 3560.}	118.312	0.255408	newly 3 weeks cumul	5250.	8909.19	{4152., 16984.}	79.834	0.202612	newly 5 weeks spec	248.	813.388	{348., 1568.}	227.979	0.415706	newly 5 weeks cumul	5990.	10961.7	{5061., 20903.}	89.9178	0.215487	newly 54 weeks cumul	6323.	12414.4	{5637., 23637.}	100.861	0.231471
49	newly 3 weeks spec	879.	822.722	{350., 1543.}	47.3644	0.220943	newly 3 weeks cumul	4780.	3894.83	{1709., 7280.}	46.5446	0.24439	newly 5 weeks spec	321.	364.056	{155., 707.}	52.1374	0.200579	newly 5 weeks cumul	5641.	4801.58	{2101., 8952.}	46.5412	0.235657	newly 52 weeks cumul	6148.	5495.85	{2422., 10310.}	46.8045	0.226915
50	newly 3 weeks spec	1513.	1794.54	{870., 2726.}	52.639	0.178046	newly 3 weeks cumul	7746.	8098.79	{4292., 12708.}	44.7875	0.178145	newly 5 weeks spec	636.	820.	{384., 1226.}	60.0379	0.180854	newly 5 weeks cumul	9438.	10137.2	{5310., 15658.}	46.7791	0.179103	newly 53 weeks cumul	10556.	11729.9	{6028., 18073.}	48.6082	0.178112
